# MEERCAT: Multiplexed Efficient Cell Free Expression of Recombinant QconCATs For Large Scale Absolute Proteome Quantification*[Fn FN2]

**DOI:** 10.1074/mcp.RA117.000284

**Published:** 2017-10-20

**Authors:** Nobuaki Takemori, Ayako Takemori, Yuki Tanaka, Yaeta Endo, Jane L. Hurst, Guadalupe Gómez-Baena, Victoria M. Harman, Robert J. Beynon

**Affiliations:** From the ‡Proteo-Science Center, Ehime University, Ehime, 791–0295, Japan;; §Advanced Research Support Center, Ehime University, Ehime, 791–0295, Japan;; ¶The United Graduate School of Agricultural Sciences, Ehime University, Ehime, 790–8566, Japan;; ‖Centre for Proteome Research, Institute of Integrative Biology, University of Liverpool, Liverpool, L69 7ZB, UK;; **Mammalian Behaviour and Evolution Group, Institute of Integrative Biology, University of Liverpool, Leahurst Campus, Neston CH64 7TE

## Abstract

A major challenge in proteomics is the absolute accurate quantification of large numbers of proteins. QconCATs, artificial proteins that are concatenations of multiple standard peptides, are well established as an efficient means to generate standards for proteome quantification. Previously, QconCATs have been expressed in bacteria, but we now describe QconCAT expression in a robust, cell-free system. The new expression approach rescues QconCATs that previously were unable to be expressed in bacteria and can reduce the incidence of proteolytic damage to QconCATs. Moreover, it is possible to cosynthesize QconCATs in a highly-multiplexed translation reaction, coexpressing tens or hundreds of QconCATs simultaneously. By obviating bacterial culture and through the gain of high level multiplexing, it is now possible to generate tens of thousands of standard peptides in a matter of weeks, rendering absolute quantification of a complex proteome highly achievable in a reproducible, broadly deployable system.

One of the major challenges in proteomics is absolute quantification of individual proteins. The predominant technology in large scale protein quantification is MS of (usually tryptic) peptides derived from proteolysis of the proteome *in vitro* and it is well understood that although mass spectrometers can deliver linearity of response over many orders of magnitude, the response factor (signal intensity per mol of peptide) varies considerably among individual peptides (1, 2). One outcome is that commonly used “label-free” methods that sum the precursor ion intensities for the peptides derived from a single protein, are excellent for relative quantification, but are less satisfactory for absolute quantification.

MS-based absolute quantification of proteins can be supported by external standards that are analyzed before and/or after the analyte or by stable-isotope labeled internal standards that are coanalyzed and which define the response factor for each peptide (3). These peptides can be individually synthesized and quantified (4) and there have been some remarkable large-scale studies. However, large numbers of accurately quantified peptides are costly. Further, a commercially produced, accurately quantified standard peptide is a finite resource and is hence best focused on low numbers of assays of a small number of target proteins. Intact protein standards (5–7), or large fragments (8) provide multiple potential peptides for quantification of the targets.

In 2005, a novel approach to the creation of standard peptides by biosynthesis was proposed in the form of QconCATs (9–13). QconCATs are artificial proteins that are concatenations of standard peptides from multiple natural proteins, sometimes interspersed by short peptides to recapitulate the primary sequence context of the natural counterpart (14, 15). Peptides suitable for quantification are referred to as Q-peptides, and are not synonymous with proteotypic peptides, as the latter term refers to peptides, unique to one protein, that drive protein identification, not quantification. QconCATs genes are synthesized *de novo* and are routinely expressed in *E. coli* cultured in media supplemented with appropriate stable isotope labeled amino acids, such that peptides derived from QconCATs are discriminable from natural peptides within the mass spectrometer. The purified QconCATs are mixed with the biological analyte sample and coproteolyzed to generate a mixture of labeled (standard) and unlabeled (analyte) peptide pairs that can be analyzed by liquid chromatography coupled to MS to yield absolute quantification of the analyte proteins. QconCATs have the added advantage that with appropriate control of proteolysis (11) all standards are, by definition, in a 1:1 ratio, rendering independent quantification of each standard unnecessary; a single common peptide can function to quantify the QconCAT (13). However, successful expression of novel QconCATs in *E. coli* is not always guaranteed. In a large-scale quantification project that used over 100 independently designed and expressed QconCATs, we discovered that ∼1 in 10 of the concatamers would fail to express, even when a range of expression conditions were explored. Further, at a low frequency, some QconCATs were prone to proteolysis in the bacterial cell or during purification, rendering them of reduced value for quantification.

Effective QconCAT deployment across large scale proteome quantification studies would require a high level of confidence in expression of every new construct. In addition, living-cell based synthesis systems are not ideal for high-throughput preparation of multiple QconCATs and many mass spectrometry laboratories are not equipped for the basic molecular biology that would be needed to subclone and express recombinant proteins. To enhance the potential of QconCAT technology for large-scale proteome quantification, we here focus on a wheat germ cell-free protein synthesis system (WGCFS)^1^ as a major enhancement to the workflow of high throughput QconCAT synthesis. WGCFS, which uses the powerful translation system for germination stored in wheat germ, realizes the highest yield of translation among commercially available eukaryotic derived cell-free systems (16–20). Using WGCFS, we previously demonstrated the feasibility of synthesis of single, small QconCATs, typically 25 kDa (21). In the present study, we first assessed whether WGCFS could be used to express more typical QconCATs at approx. 60 kDa (for quantification of ∼25 proteins at two peptides per target protein), whether WGCFS would rescue “failed” QconCATs and whether this cell free system was able to reduce the risk of proteolytic degradation. Further, we established whether an additional step in efficiency could be derived from coexpression of multiple QconCATs in a single WGCFS reaction.

## EXPERIMENTAL PROCEDURES

### 

#### 

##### Subcloning of QconCAT Genes

From the QconCAT gene library based on plasmid vector pET21a (University of Liverpool) (13), twelve QconCATs (one that expressed well: CC# 001, and eleven that had failed to express: CC# 024, 039, 046, 057, 062, 064, 066, 068, 069, 099, and 103) were selected for subcloning into the cell-free expression vector pEU-E01-MCS (CellFree Sciences, Matsuyama, Japan). Target QconCAT gene was amplified by PCR using a sense primer (5′-ATATACATATGGCAGGTCGTG-3′) and an antisense primer (5′-CCGGGATCCCCATCATTAGTG-3′). The obtained PCR products were ligated with the EcoRV and BamHI sites of the pEU-E01-MCS. The ligation products were then transformed into the *E. coli* DH5α. After overnight culture in LB medium containing 50 μg/ml ampicillin, plasmid DNA was purified with the alkaline lysis method. Correct insertion of the QconCAT genes into the pEU-E01-MCS was verified by DNA sequencing.

##### DNA Template Preparation for In Vitro *Transcription Using PCR*

PCR-based cell-free expression templates were prepared using a split-primer PCR method (18). The first PCR amplification was performed using a sense primer (5′-CCACCCACCACCACCAATGGC[A/C/G/T]GG[C/T]CGTGA[A/G]GG[C/T]G-3′) and an antisense primer (5′-CTTTTCTACGGGGTCTGACG-3′). In the second PCR amplification, following primers were used: SPu primer (5′-GCGTAGCATTTAGGTGACACTATAGAAC-3′), deSP6E01 primer (5′-GGTGACACTATAG-AACTCACCTATCTCCCCAACACCTAATAACATTCAATCACTCTTTCCACTAACCACCTATCTACATCACCACCCACCACCACCAATG-3′), and the antisense primer. The second PCR products were purified with ethanol precipitation and resolubilized with nuclease-free water. The quality of PCR generated template was assessed by agarose gel electrophoresis. DNA concentrations were determined using Qubit® DNA Assay Kits (Life Technologies, Carlsbad, CA, USA) according to the manufacturer's instructions.

##### Cell-free Synthesis of Stable Isotope Labeled QconCATs

For cell-free synthesis of QconCATs, we used the WEPRO 8240 H (K; ^13^C,^15^N, R; ^13^C,^15^N) Expression kit (Kit No. 10FX01–12B03) from Cell Free Sciences, Matsuyama, Japan. In the wheat germ cell-free synthesis experiment, transcription and translation reaction are performed independently (22) and the mRNA used for cell-free synthesis was prepared by *in vitro* transcription reaction using SP6 RNA polymerase for 6 h at 37 °C. Translation *in vitro* of heavy-labeled QconCAT proteins, using the bilayer method (20, 21), was for 20 h at 16 °C. In small-scale synthesis experiments, 200 μl substrate layer and 40 μl translation layer were prepared in a 96-well microplate. In large-scale cosynthesis experiments, translation *in vitro* was in a 6-well microplate (1 ml translation layer and 5 ml substrate layer) or a 12-well microplate (0.2 ml translation layer and 1 ml substrate layer). For high efficiency stable-isotope labeling, [^13^C_6_,^15^N_4_]Arg and [^13^C_6_,^15^N_2_]Lys (Wako, Osaka, Japan) were added to the translation layer (final concentration of 20 mm each). The detailed procedures are shown in supplemental Fig. S1.

##### Purification of Synthesized QconCAT Proteins

After completion of cell-free synthesis, 200 μl of the crude cell-free reaction mixture was diluted with an equal volume of binding buffer (20 mm sodium phosphate, 0.5 m NaCl, 10 mm imidazole, pH 7.4) and incubated with 10 μl of Ni-Sepharose High Performance resin (GE Healthcare, Pittsburgh, PA, USA) on a rolling wheel for 1 h at room temperature. The resin was washed twice with 400 μl of wash buffer (20 mm sodium phosphate, 0.5 m NaCl, 50 mm imidazole, pH 7.4). Binding proteins were eluted with 30 μl of an elution buffer (20 mm sodium phosphate, 0.5 m NaCl, 500 mm imidazole, pH 7.4).

##### Tryptic Digestion of Gel-separated QconCATs

Synthesized and purified QconCAT proteins were separated on a NuPAGE 4–12% Bis-Tris gel (Life Technologies), and stained using Bio-Safe CBB (Bio-Rad, Hercules, CA). Protein bands were excised from the gel, and each gel piece was destained using 50% (v/v) acetonitrile in 100 mm ammonium bicarbonate. After washing with 100 mm ammonium bicarbonate, proteins were reduced with 10 mm dithiothreitol for 1.5 h at 37 °C, and subsequently alkylated with 50 mm acrylamide for 30 min at room temperature. Proteins in the gel were digested with sequencing-grade modified trypsin (Promega, Madison, WI) at 37 °C overnight.

##### Mass Spectrometry

Tandem MS (MS/MS) and selected reaction monitoring (SRM) was on an Eksigent nanoLC system connected to a Q-Trap 5500 mass spectrometer (SCIEX, Framingham, MA, USA) as described previously (21). The digested peptide samples were injected onto a 200 μm ID × 0.5 mm cHiPLC trap column (SCIEX). Concentrated peptides were then separated on a 75-μm ID × 15 cm C18 reversed phase cHiPLC column (SCIEX). Solvent A was 0.1% formic acid in water. Solvent B was 80% acetonitrile containing 0.1% formic acid and water. The flow rate was set to 300 nL/min. The following gradient was used: 0–30 min, 2–30% B; 30–35 min, 30–90% B; hold at 90% B for 10 min, and equilibrate at 2% B for 15 min prior to next run. For protein identification, obtained MS spectra were searched by the ProteinPilot database ver.4.0 (SCIEX) using the following parameters: cys alkylation, acrylamide; digestion, trypsin; processing parameters, biological modification; and search effort, through ID.

For global proteome analysis, the protein concentration of all samples for data directed acquisition (DDA) was determined using a NanoDrop™ spectrophotometer. All samples were diluted appropriately in 25 mm ammonium bicarbonate for each digest volume of 50 μl to contain 25 μg protein. Samples were treated with RapiGest™ at 80 °C for 10 min and proteins were reduced with 3 mm DTT at 60 °C for 10m, alkylated with 9 mm IAA at room temperature in the dark and digested with a 50:1 ratio to Trypsin Gold (Promega). Digestion was overnight at 37 °C. The resulting digest was acidified with 1% (v/v) final concentration of trifluoracetic acid for 45 min followed by centrifugation at 16,000 x *g* for 30min. Peptides were analyzed on a Thermo QExactive HF using a one hour gradient. The resulting raw files were processed using Progenesis (ver 3.0) and searched using Mascot (ver 2.6) against a database containing the 75 QconCAT sequences (provided as supplementary Data File D1) included in the synthesis with fixed acrylamide modification of cysteine residues, variable Met oxidation, and for labeled samples, variable labeling with [^13^C_6_,^15^N_4_]Arg and [^13^C_6_,^15^N_2_]Lys. Label free quantitative values were obtained from MS1 signal intensities summed for all unique peptides.

##### Absolute Quantitation of QconCATs

A variant of Glu-fibrinopeptide (FP) (GVNDNEEGFFSAR), released from the C terminus of the QconCATs by trypsin, was used for the absolute quantification of synthesized QconCATs using MS. Synthetic unlabeled FP (Sigma-Aldrich, St. Louis, MO), which was quantified by amino acid analysis, was used as an internal standard. The QconCAT sample dissolved in 0.1% (w/v) RapiGest surfactant (Waters, Milford, MA) was subjected to trypsin digestion after adding internal standard. After digestion at 37 °C for overnight, samples were mixed with an equal volume of 1% (v/v) trifluoracetic acid and incubated at 37 °C for 10 min. After centrifugation at 18,000 × *g* for 3 min, the supernatant was subjected to STAGE tip purification. The FP present in the purified peptide sample was measured by SRM. The amount of synthesized QconCAT was estimated based on the peak area ratios of the heavy and the light (unlabeled internal standard) forms of FP.

## RESULTS

**Fig. 1.**
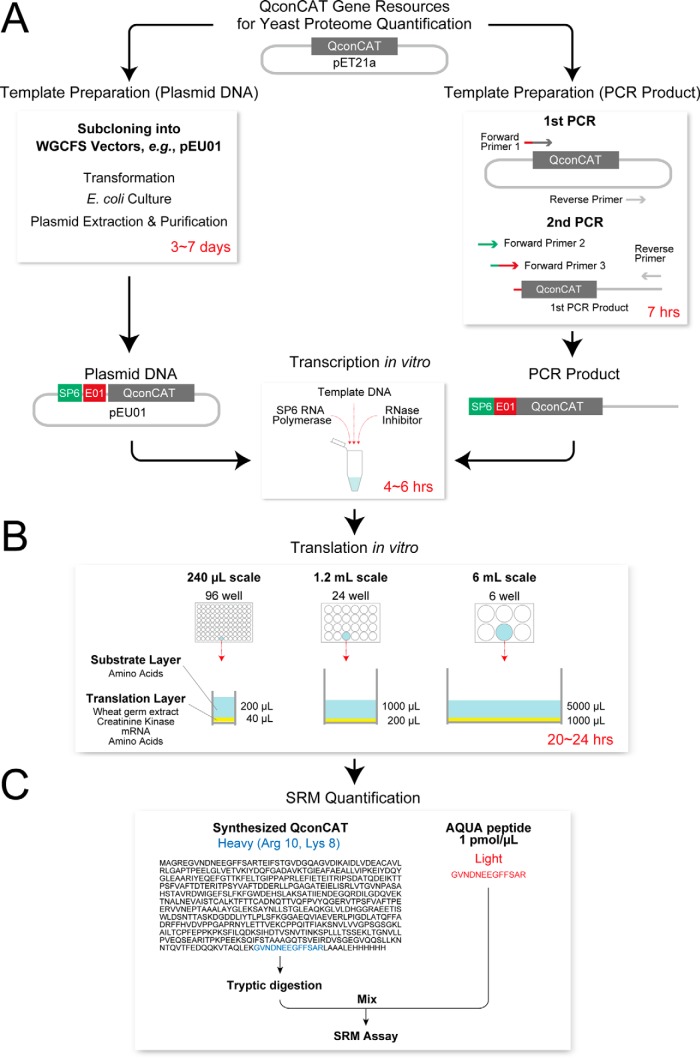
**Overall experimental design for QconCAT expression in a cell-free system.** Existing QconCAT genes, cloned into pET-21a, were recovered by a two stage PCR process to create PCR products that contained both the SP6 promoter and the E01 translation enhancer (panel *A*). The PCR products were then used to first direct RNA synthesis *in vitro*, and the transcripts were used without purification in the cell-free translation reaction, initially in 240 μl synthesis reactions in a 96 well plate (panel *B*). The yield of each stable isotope labeled QconCAT was measured by mass spectrometry, making use of the common glu-fibrinopeptide contained in each QconCAT and accurately quantified unlabeled glu-fibrinopeptide as a standard (panel 1C).

Cell free synthesis can be initiated with either amplified PCR products or plasmid “mini-preps” with no effect on yield of output. Either a linear PCR product or circular plasmid DNA is first used to direct synthesis of the appropriate RNA, synthesized by an SP6 RNA polymerase. The RNA contains an E01 translation enhancer sequence. DNA templates were obtained by amplification of the QconCAT sequence from the existing plasmids by PCR, followed by a second, “split primer” PCR reaction to create QconCATs preceded by the SP6 and E01 sequences. These PCR products were then used as the template in the transcription reaction. The overall workflow is described in Fig. 1.

### 

#### 

##### WGCFS Can Rescue QconCATs That Do Not Express In Vivo

**Fig. 2.**
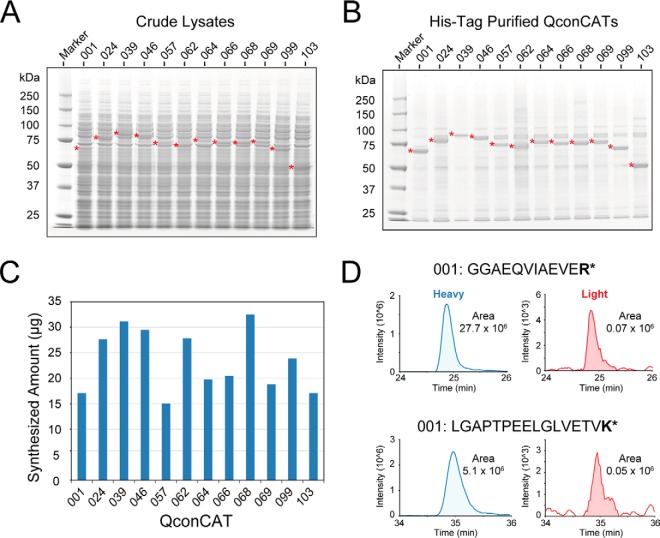
**Expression *in vitro* of multiple QconCATs.** Twelve QconCAT genes, eleven of which had failed to express *in vivo* were selected. The coding regions was recovered by selective PCR amplification (panel *A*) and were expressed *in vitro* by cell-free synthesis in the presence of [^13^C_6_][^15^N_4_]arginine and [^13^C_6_][^15^N_2_] lysine and purified by a hexahistidine tag (panel *B*). The QconCATs were quantified by selected reaction monitoring using Glu-fibrinopeptide as standard (panel *C*), indicating typical yields of 250 pmol per reaction. Labeling of peptides was extremely efficient (panel D).

From the QconCAT series developed for large-scale quantification of the *Saccharomyces cerevisiae* proteome (11, 13), we first selected a subset of 11 QconCAT genes that had failed to express in *E. coli* along with one well expressed QconCAT (Protein sequences are presented in supplementary Data File D1). After subcloning the QconCAT genes into a plasmid vector optimized for WGCFS, a set of small-scale synthesis reactions (240 μl) was conducted. On SDS-PAGE of the total WGCFS reaction mixture, all QconCATs were detectable as the strongest band at the expected mobility on the Coomassie-stained image (Fig. 2*A* prepurification; Fig. 2*B*, postpurification), clearly visible above the WGCFS background. After trypsin digestion and analysis by liquid chromatography-mass spectrometry (LC-MS), all but five of 556 tryptic Q-peptides were detectable by tandem mass spectrometry (supplemental Fig. S2). In all instances, N and C-terminal peptides were detectable, additional evidence for expression of full-length QconCATs. The total synthesis, assessed by a common quantification tag (GVNDNEEGFFSAR), was of the order of low tens of micrograms (23 ± 6 μg, *n* = 12) or 317 ± 69 pmol (mean ± S.D., *n* = 12, Fig. 2*C*, supplemental Fig. S3). The yield was thus sufficiently high to support hundreds or thousands of selected reaction monitoring (SRM) assays, each of which would typically require 0.1 to 10 fmol “on column” in a typical LC-MS/MS experiment. Further, the isotopic labeling efficiency of synthesized QconCATs was extremely high, averaging 99.6% (Fig. 2*D*, supplemental Table S1). This is more labeling than obtained during expression *in vivo* in *E. coli*, presumably because there was no biosynthesis of unlabeled amino acid to reduce the relative isotope abundance of the precursor. More extensive labeling extends the dynamic range for quantification as it reduces the contamination of the “light” channel by unlabeled material from the partially labeled “heavy” peptide. Particularly noteworthy was the ability of WGCFS to express high levels of every QconCAT that had failed to express in *E. coli*. Expression *in vitro* precludes issues of cytotoxicity and it is highly probable that all QconCATs will be readily expressed in future, further enhancing the efficiency of generation of these standards.

##### WGCFS Can Reduce Proteolytic Damage to QconCATs

**Fig. 3.**
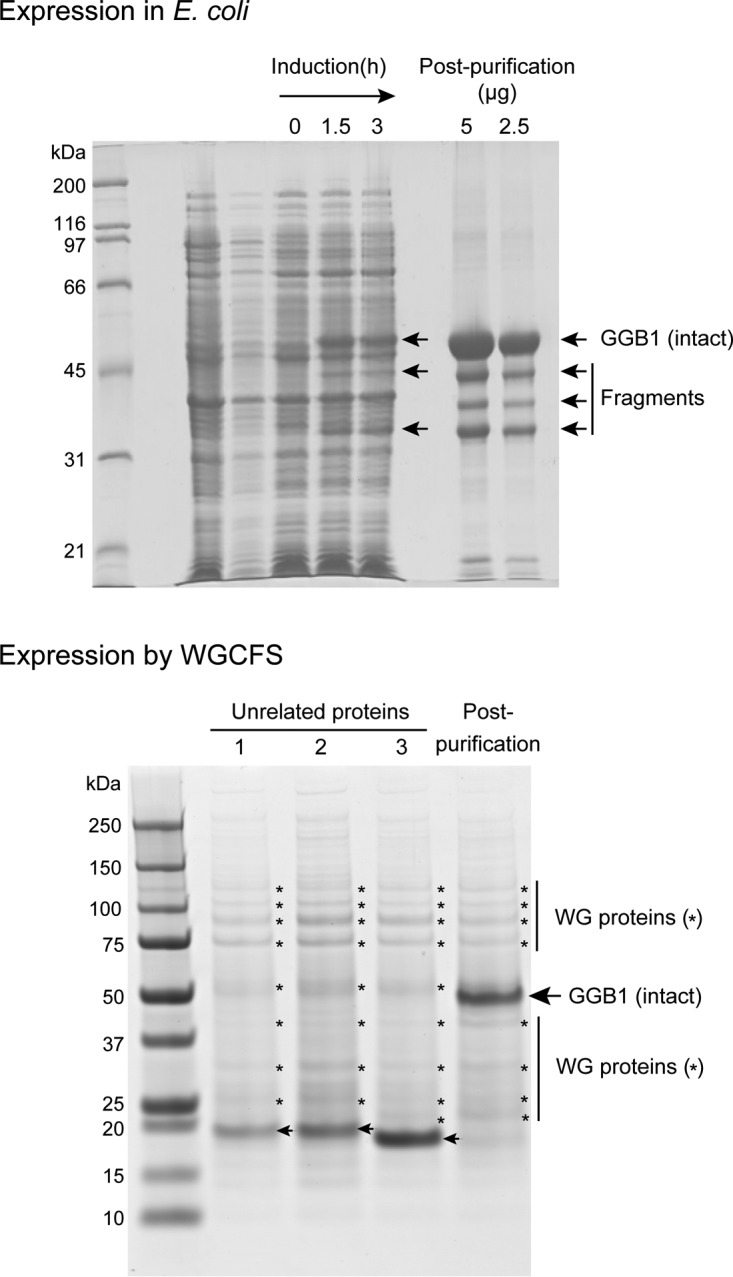
**Prevention of proteolysis of QconCATs in the cell-free expression system.** One QconCAT (GGB1, for quantification of isoforms of major urinary proteins) was expressed in *E. coli*. During expression, and after histag purification, several low molecular weight species products were copurified (“Fragments”) These were demonstrated to be fragments of GGB1 by in gel digestion and mass spectrometry. When expressed in the cell-free system, no proteolytic degradation products were apparent. The bands in the background marked with an asterisk were derived from the cell-free system, further evidence by their appearance in the products of three unrelated, low molecular weight proteins (highlighted by arrows at 20 kDa).

When expressed in *E. coli*, QconCATs are predominantly expressed in insoluble form and accumulate in inclusion bodies, which protects them from intracellular degradation. However, on occasion, we have noted that a purified QconCAT has undergone marked proteolytic damage, which would make it difficult to use in a quantitative analysis. One of the features of the WGCFS is the low level of endogenous proteases resulting from the thorough removal of endosperm components (17), reducing the chance of degradation. We therefore expressed, in the WGCFS, a QconCAT that was known to undergo proteolysis in *E. coli*. High yields of this protein were obtained, and the protein was intact and of the predicted mobility on SDS-PAGE, with no evidence of degradation (Fig. 3). The wheat germ system is likely to be able to protect QconCATs from proteolytic degradation which, even if only partial, could compromise the stoichiometric equivalence of each standard peptide embedded within the concatamer.

##### WGCFS Allows Coexpression of Multiple QconCATs

**Fig. 4.**
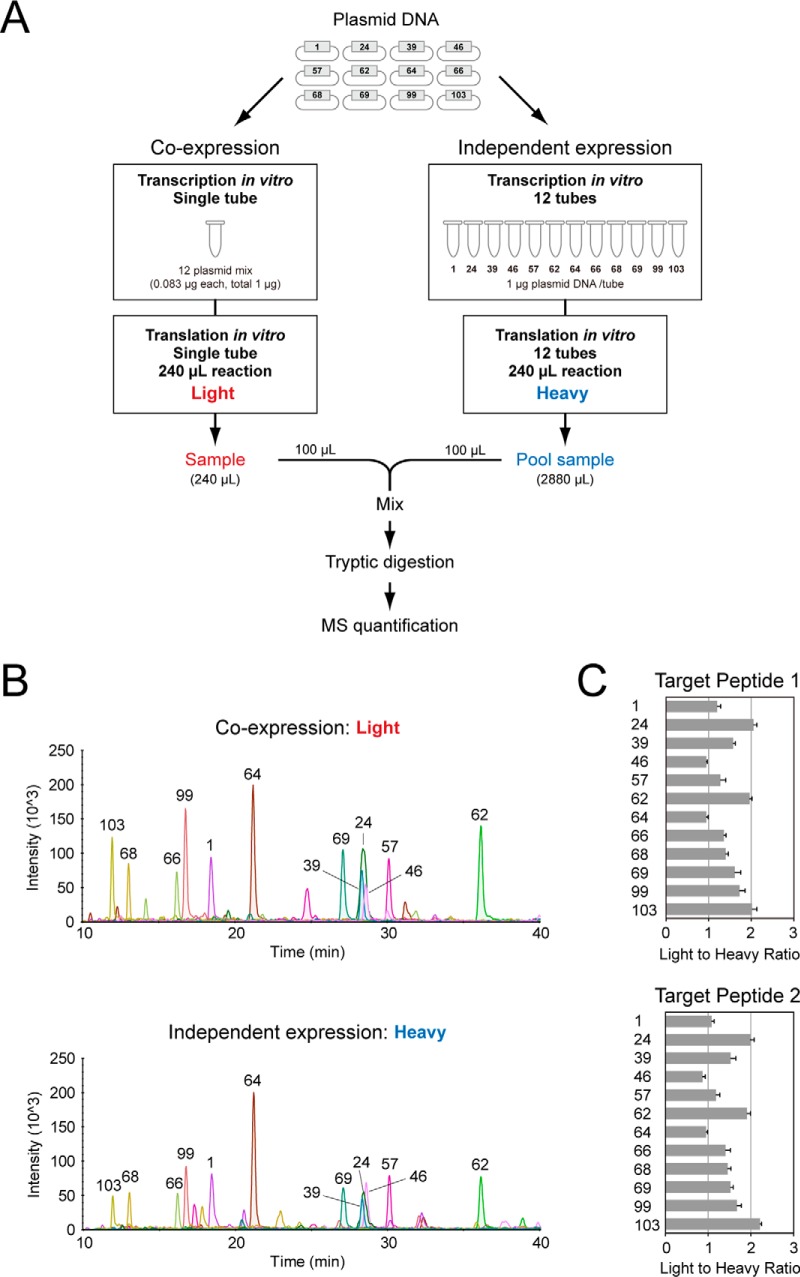
**Multiplexed expression and quantification of QconCATs.** Twelve QconCATs were either independently synthesized and labeled with [^13^C_6_][^15^N_4_]arginine and [^13^C_6_][^15^N_2_]lysine or coexpressed in a single cell-free reaction without stable isotope labels (panel *A*). After both biosynthetic reactions were completed, the reaction mixtures for the individually synthesized QconCATs (each 240 μl) were pooled. This pool was then mixed with an equal volume of the cosynthesis reaction mixture and the relative yield of each QconCAT was assessed by SRM assays to permit determination of the heavy:light ratio for each SRM peptide (panels *B* and *C*) using two peptides for each QconCAT, described in supplemental Fig. S4.

Protein synthesis *in vitro*, more flexible than protein synthesis *in vivo*, can allow simultaneous translation of multiple genes in a single reaction (23–27). It follows that multiple QconCATs could be coexpressed in a single lysate, opening the possibility of an efficient route to highly multiplexed, absolute quantification of proteomes, bringing closer global proteome quantification. We therefore conducted a cosynthesis experiment of the same 12 QconCATs (Fig. 4*A*), each of which was capable of being synthesized in independent reactions (as described in Fig. 1). The cosynthesis mixture of QconCATs was expressed in the absence of stable isotope labels, and at the end of the reaction the production of each QconCAT was evaluated by SRM using accurately quantified, equivalent QconCATs, synthesized independently with stable isotope labeled lysine and arginine in the reaction mixture (Fig. 4*B*). Comparison of the “light” (cosynthesis) and “heavy” (independent synthesis) channels would therefore indicate the impact of coexpression on the yield of all or individual QconCATs (Fig. 4*C*). Despite the complexity of the synthesis mixture, all 12 QconCATs were expressed simultaneously in a single WGCFS reaction (Fig. 4*B*, 4*C*). The yield of each QconCAT matched or exceeded that of the independently synthesized proteins (Fig. 4*C*, supplemental Fig. S4). Thus, all QconCATs were synthesized at least as well or better than individual proteins. A single reaction mixture of 240 μl, deliverable in less than 2 days, was therefore capable of supporting the synthesis of 12 QconCATs at a yield of 37 ± 12 pmol (mean ± S.D., *n* = 12, range 21 to 53 pmol) permitting hundreds of assays for the quantification of (in this example) 253 target proteins at a redundancy level of two Q-peptides for each target protein. Although the yield of each QconCAT in the cosynthesis reaction on a small scale (240 μl) was reduced when compared with independent synthesis, this was anticipated from the design, that delivered the same total DNA input, but distributed equally over 12 QconCATs (supplemental Fig. S4). In practice, the yield for most QconCATs was reduced by a lower degree than anticipated, meaning that the efficiency of cosynthesis was higher. For convenience, we refer to this approach of cosynthesis of multiple QconCATs by the acronym “MEERCAT” (Multiplexed Efficient Expression of Recombinant QconCATs) for brevity. The decreased output of each QconCAT in a cosynthesis mixture presumably reflects competition among the different QconCAT transcripts for access to the WGCFS translational apparatus, or depletion of precursors or ATP. Such limitations are readily addressed by adjustment of the scale of the biosynthesis.

##### Independent Quantification of QconCATs Coexpressed in WGCFS

**Fig. 5.**
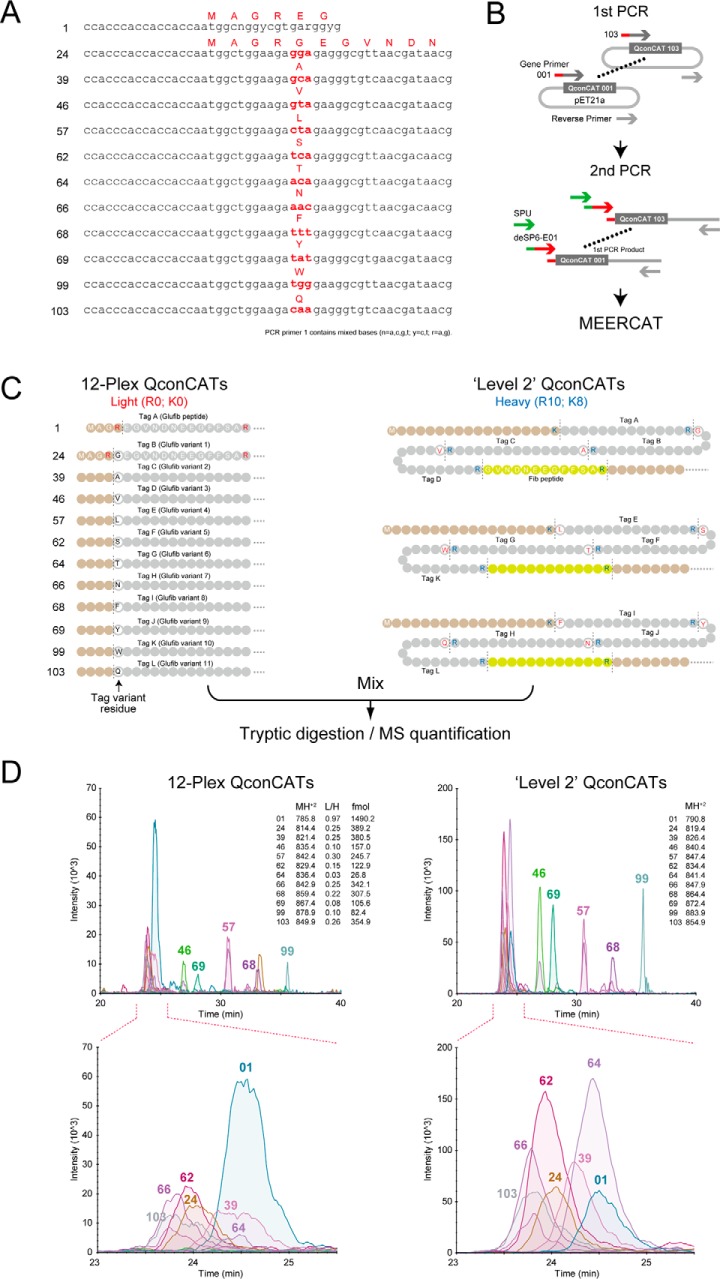
**Use of mass encoded tags for quantification of cosynthesized QconCATs.** The same twelve QconCATs described in Fig. 1 were recovered from plasmids by PCR using a series of primers that introduced variants of the N-terminal Glu-fibrinopeptide standardization peptide (panels *A*, *B*). These variant tag QconCATs were coexpressed in the absence of stable isotope labeled amino acids. Three “level 2′ QconCATs, encoding all of the tags, were also constructed, expressed and labeled with [^13^C_6_][^15^N_4_]arginine and [^13^C_6_][^15^N_2_]lysine, quantified and mixed with the unlabeled cosynthesis reaction. The mixture was digested with trypsin and analyzed by SRM (panel *C*). The variant quantification tags eluted in a tight group, readily discriminable by mass and permitting independent quantification of each coexpressed QconCAT (panel *D*).

Coexpression of QconCATs by MEERCAT would introduce the requirement that each concatamer be quantified independently, before being deployed in quantitative proteomics experiments. This would require each QconCAT to contain an independent quantification tag. As proof of concept and to illustrate the feasibility of such an approach, we created a series of mass-coded tags based on glu-fibrinopeptide (EGVNDNEEGFFSAR) lacking the N-terminal glutamic acid residue (Fig. 5*A*) by mismatch in the PCR primers. A series of variants of this peptide, differing by the addition of a single amino at the amino terminus (R/**X**EGVNDNEEGFFSAR/, in which **X** was one of G, A, V, L, S, T, N, F, Y, W or Q) were thus introduced to the set of QconCAT sequences at the N terminus by variation in the PCR primer (Fig. 5*B*), providing a mass unique independent tag for each protein. These 12 QconCATs were then expressed in a stable isotope labeled 12-plex reaction mixture. At the same time, a further three “level 2” QconCATs, encoding all variants of the independent tags were synthesized in unlabeled form and independently quantified (Fig. 5*C*). The 12-plex mixture of tagged QconCATs was then mixed with a pool of the three “level 2” standard QconCATs that had been independently quantified using a second Glu-fibrinopeptide quantification peptide. After codigestion, each of the independent variant tags gave sharp chromatographic peaks that were distributed, as expected, over a narrow range of retention times (Fig. 5*D*) and which yielded well behaved precursor and product ion spectra. Thus, independent quantification of each of the 12 products of the cosynthesis was feasible using a simple mass-coded tag.

##### Highly Multiplexed Coexpression of QconCATs

**Fig. 6.**
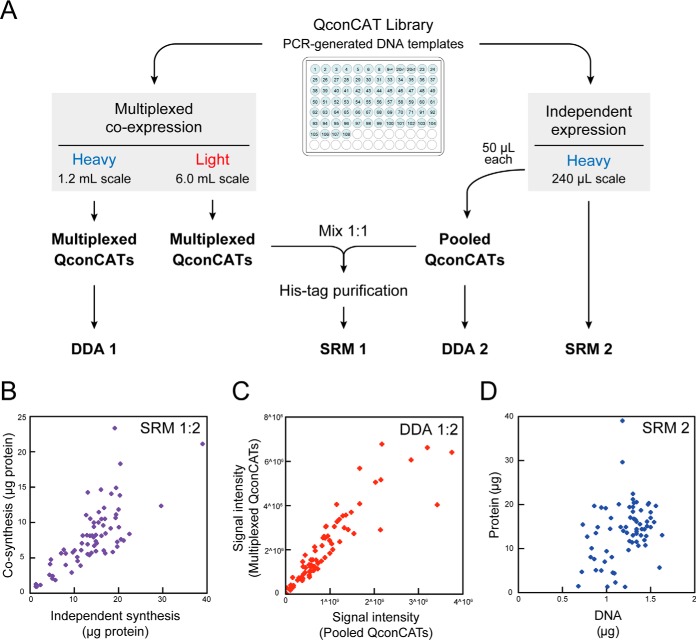
**Simultaneous coexpression of large numbers of QconCATs.** A set of 76 QconCATs were either expressed individually or in a cosynthesis multiplexed reaction (panel *A*). The individual QconCAT reaction mixtures were pooled, and the yield of QconCATs was either assessed by SRM using the Glu-fibrinopeptide standard or by data dependent, label-free quantification (DDA, based on summed MS1 intensity of unique peptides). The yield of the individual QconCATs was strongly correlated between the two synthetic approaches (panel *B*, *C*). The yield of QconCATs, assessed by SRM analysis of the individually synthesized proteins, was also correlated to the quantity of DNA that was used to drive cell-free expression (panel *D*).

Having demonstrated that 12 QconCATs (eleven of which had previously failed to express in *E. coli*) could be both rescued and expressed simultaneously *in vitro*, we then tested the ability of the system to extend the degree of multiplexing and cosynthesize a very large number of QconCATs simultaneously, in a single reaction. We therefore attempted the coexpression of a large number of “typical” 70–75 kDa QconCATs (Fig. 6*A*). We used template DNA amplified from 76 QconCAT genes previously designed for quantification of the *S. cerevisiae* proteome (13), encoding ∼4000 standard peptides. First, we established that the QconCATs could be expressed independently in low volume WGCFS experiments (supplemental Fig. S5). Of the 76, 71 were readily detectable by mass spectrometry, the low levels of the reminder we attribute to the reduced quantity of DNA available for transcript generation for that construct (see below). Then, all the QconCAT PCR products were mixed in equal volumes to create a pool of templates to direct multiplexed transcription, followed by cell-free translation. Using a larger volume for the WGCFS reaction, the average yield of QconCATs was 8 ± 4 μg (mean ± S.D., *n* = 71, range 1 to 23 μg) or an average of ∼100 pmol of each QconCAT. Whether assessed by SRM (Fig. 6*B*) or label free quantification using DDA (Fig. 6*C*), there was a strong relationship between the quantity of QconCATs produced in the multiplexed cosynthesis and the quantity from independent synthesis, implying a common factor determining yield. This might be in part attributed to translational efficiency but there was a weak correlation between the yield of each QconCAT and the amount of DNA that was used as template (Fig. 6*D*). Normalization of input DNA should be able to redress inequalities of QconCAT expression. The WGCFS clearly has the capacity for very high level multiplexed production of QconCATs in quantities appropriate to quantitative proteomics studies. The limits of the number of genes that can be synthesized simultaneously in WGCFS will be further expandable. In fact, simultaneous synthesis of 150 small (25kDa) QconCATs (21) was possible in a 6-ml reaction volume, of which 149 QconCATs expression was confirmed by MS/MS (supplemental Fig. S6). However, because the resources required for synthesis in the reaction solution are finite, further increase in cosynthesis number is expected to lead to a decrease in the expression yield of individual QconCAT. When considering the use as an internal standard in mass spectrometry, the optimal number of QconCATs that can be cosynthesized will be between 50 and 100.

## DISCUSSION

### 

#### 

##### Performance and Future Applications of MEERCAT

Sophisticated systems modeling of biological processes requires accurate, absolute abundances of proteins. These data are remarkably nontrivial to acquire. Moreover, technical approaches are not consistent in the quantification values that they generate. The most robust and unbiased method is based on LC-MS, supported by accurately quantified standard peptides that are coanalyzed and discriminated by the mass offset derived from incorporation of stable isotope atom centers. The largest absolute quantification study completed to date has been for ∼1900 proteins in *S. cerevisiae* using the QconCAT strategy (13), a program that took several years. In addition to providing a gold standard of accurately quantified yeast proteins, this study also revealed the challenges inherent in this approach, including a significant rate of quantitative discordance between two peptides targeted to a single protein and the finite, albeit low, rate of failure of QconCAT expression. For absolute quantification to become more accessible and broadly feasible, the need for more efficient synthesis of concatenated standards became evident during this study.

WGCFS showed high expression capability in the synthesis of artificially designed QconCATs. Because WGCFS does not require codon optimization, the QconCAT sequence constructed for expression in *E. coli* could also be used unchanged. This result strongly suggests the possibility that many QconCAT resources developed so far can be smoothly transferred to WGCFS. In this study, the cell-free synthesis of QconCAT was performed using the bilayer reaction method (22). This method enables high-efficiency translation reaction for 24 h by separating the substrate layer and the translation layer. In addition, highly efficient incorporation (>99%) of stable isotope into synthetic proteins can be easily achieved by adding stable isotope-labeled amino acids of >20 mm to the translation reaction layer (20). We have demonstrated that the independent synthesis or cosynthesis reaction by the bilayer method could provide sufficient yield of stable isotope-labeled QconCATs for MS analysis within 20–24 h (Figs. 2, 4). A further increase in yield is obtained by the introduction of a dialysis method which allows continuous reaction, but in most cases, it will be unnecessary. Therefore, we concluded that low cost and simple bilayer system is appropriate for MEERCAT. The synthesis reaction on a small scale of several hundred microliters can reduce the amount of expensive stable isotope-labeled amino acids to be added while ensuring sufficient QconCAT synthesis amount for mass spectrometry, which greatly contributes to a cost reduction of standard synthesis for absolute quantification. If PCR-based template construction was performed instead of time-consuming subcloning, one experimenter could fully synthesize more than 50 QconCATs during 2 days, as demonstrated here.

We have demonstrated the simultaneous biosynthesis of large numbers of QconCATs, an approach that greatly enhances the opportunities for large scale absolute proteome quantification. Coexpression of recombinant proteins in living cells tends to elicit extreme bias in their expression levels (24). On the other hand, in a cell-free synthetic system, cosynthesis reaction with relatively little bias is possible (24, 25), and it is easy to scale up the number of genes used for coexpression (27). In this study, we have succeeded in creating the largest scale cell-free cosynthesis reaction to date and obtain sufficient expression level for MS analysis. Interestingly, there was no bias in the expression level of each QconCAT in the cosynthesis reaction as compared with independent synthesis (Fig. 4). In the cell-free synthesis system of *E. coli*, homogenization of expression level can be obtained by retaining identical 5′ terminal sequences in each gene (25). Because QconCATs used this study have a consensus sequence at the N terminus, there is a high possibility that the same effect was obtained even in WGCFS. Introduction of a cosynthesis strategy can achieve significant reduction in the cost and feasibility of preparation of internal standards. Ten WGCSF reactions, each coexpressing 100 QconCATs could realize the simultaneous synthesis of 1000 QconCATs. At a typical size of 50 Q-peptides per QconCAT, and a quantification ratio of two peptides per target analyte protein, this would yield a resource for quantification of 25,000 proteins, enough to cover the human proteome. These 10 reactions could be conducted in parallel in 2 to 3 days, starting from a panel of plasmid DNA. Because the SP6 transcription reaction can use PCR products or plasmid DNA as template, the route to delivery of large quantities of templates, through plasmid “minipreps,” and hence transcripts, is straightforward. In future designs, the SP6 and E01 sequences would be engineered into the initial DNA constructs, or obtained from a suitable plasmid, such as pEU-01, that contains both leader sequences within the plasmid. Thus, a panel of QconCAT plasmids would be readily propagated and distributed. This would be a remarkable gain in the delivery of stable isotope standards and renders other approaches (individual QconCATs, PSAQs and AQUA peptides) slow and expensive by comparison.

There remains the challenge of obtaining accurate quantification of each QconCAT in the reaction mixture. We have demonstrated that it is feasible to create subtly variant quantification tags by single amino acid modifications of a standard peptide. The same tags can of course be re-used, because there is no feasible route to the simultaneous absolute quantification of an entire proteome in a single LC-MS analysis - repeated analyses using the same sets of QconCATs would be necessary. As an alternative to development of a panel of mass-coded tags, equally feasible would be the hierarchical quantification approach (28), in which unlabeled QconCATs, containing one peptide from each QconCAT in the WGCFS cosynthesis, would be prepared in a separate reaction in unlabeled form. However, this approach precludes simultaneous quantification of standard and biological analyte, and there is undoubtedly scope for development of a series of novel peptides that would allow both standard and biological analyte to be quantified in the same reaction. We would thus propose a more robust approach based on use second order “level 2” QconCATs, similar to the approach we adopted with the 12-plex reaction (supplemental Fig. S7). We propose construction of 10 distinct and re-useable tag sequences (1–10), each of which further encodes modest amino acid variation (A-J, 10 variant positions per peptide). Each QconCAT would contain then contain a single tag, and allow encoding of 100 proteins for quantification. Thus, the first peptide would be encoded with Tag1A. The second QconCAT would be differentially identifiably by Tag1B, the 11^th^ QconCAT would be encoded by Tag2A, the 12^th^ QconCAT by Tag2B, the 100^th^ QconCAT by Tag10J. For quantification, we would then express and accurately quantify second order QconCATs, optimized for rapid and effective proteolysis and including all 100 Tags. Moreover, careful analysis of the relative signal intensities of the tags, optimally selected from 16 amino acids, would permit a cross check of expression from the relative intensities in a preliminary MS analysis, allowing rapid progression to true quantification experiments. For smaller projects, it might be more efficient to continue with WGCFS expression of individual QconCATs, followed by purification, independent quantification and pooling for downstream biological projects.

It is reasonable to explore the relative cost of traditional QconCAT biosynthesis with the MEERCAT, starting in each instance with the synthesized DNA. For a single QconCAT, transformation, colony picking, growth, expression and purification of the QconCAT would take 9 days. By contrast, WGCFS production of the QconCAT (transcription, translation and QconCAT purification would be completed in 3 days. However, the gains are even more apparent when comparing expression of 12 QconCATs, for example. The cost of the DNA is similar, but for MEERCAT, all plasmids can be transcribed and translated in the same reaction, and the overall time is no different to that for a single QconCAT. For individual bacterial QconCAT expression, it is likely that no more than 6 QconCATs could be processed simultaneously, and thus, the time required for expression is substantially greater (approximately twenty days compared with 3 days, a 7-fold saving in staff time). We have also calculated the relative reagent costs for the two approaches. A single QconCAT prepared by bacterial expression would cost twice as much as a single MEERCAT reaction and both scale linearly with the number of QconCATs. A major difference between the two approaches is the final yield of purified standard; the cell culture method can yield 1–5 mg of QconCAT, but, as reported here, a low volume MEERCAT reaction yields 25 μg of QconCAT. We do not consider that this is major limitation, as the lower quantity would still be adequate for hundreds of quantification analyses. Standards that would be used regularly could be readily expressed by MEERCAT in larger volumes, although the cost would rise proportionately with the larger volume of the WGCFS. Ultimately, any increase in cost of MEERCAT would be more than compensated by the ∼7-fold saving in staff time, however. The relative workflows, starting from available DNA, are summarized in supplemental Fig. S8.

The remarkable jump in efficiency gained by this approach could transform global quantitative proteomics. To be able to generate a complete series of standards that could be prepared locally, in adequate quantities and suitable for SRM, SWATH or PRM analyses would obviate the need for molecular biology skills in proteomics labs, and would be highly efficient. Quantification kits could also be developed to target specific pathways or panels of biomarkers. A higher Q-peptide:target protein ratio could quantify post-translational variants by quantifying total protein and subtraction of the unmodified fraction defined by a second PTM-targeted site (29). Further gains in efficiency will derive from the avoidance of failed QconCATs expressed *in vivo* and the reduction of risk of proteolytic damage post expression. In QconCATs, the Q-peptides that are used do not have to be contiguous in the target protein. This might alter the primary sequence hydrolysis rate of the standard and analyte, but quantification reactions should be stoichiometrically matched (taking both reactions to completion) rather than kinetically matched (assuming identical rates of hydrolysis). The rates of hydrolysis can be equalized by inclusion of interspersed sequences that mimic the natural primary sequence context (as in (15)). There is an inherent risk in adopting a “kinetic model” and assuming that a concatenated standard and the native protein would exhibit the same digestion kinetics, even if the primary sequence context is identical. This applies to AQUA and PSAQ quantification as much as QconCAT; because it cannot be assumed that analyte and standard will be digested at the same rate, it is necessary for both reactions to proceed to completion. For a highly multiplexed MEERCAT reaction, it is likely that there would need to be some control over the quantity of each QconCAT that was to be synthesized, to better match the natural dynamic range of the target proteins. This can be achieved by adjustment of the DNA in the transcription step, rather than subsequent manipulation of the transcript that is more prone to degradation. Alternatively, different QconCATs could be coexpressed and pooled in an abundance matching strategy (see below).

A second factor in proteome quantification relates to the analytical load of large scale quantification experiments using these different approaches. With data independent acquisition methods, or fast triple quadrupole instruments, it is possible to complete the absolute quantification of many hundreds of proteins in single LC-MS/MS runs. The analysis would therefore include standards for the same number of proteins. At the high levels of multiplexing offered by QconCAT based approaches, 10 QconCATs mapping 250 proteins would introduce about 500 analyte peptides, which, in a complex, cell-derived protein samples, would comprise at least 50,000 measurable, high abundance analyte peptides. By contrast, the addition of 250 intact protein standards would expand the peptide pool by perhaps 6000 peptides, creating a substantial additional load on the duty cycle of modern mass spectrometers. Thus, a MEERCAT approach would create much less of a load on the analytical capacity of the LC-MS/MS system.

##### A “Human MEERCAT” Project

**Fig. 7.**
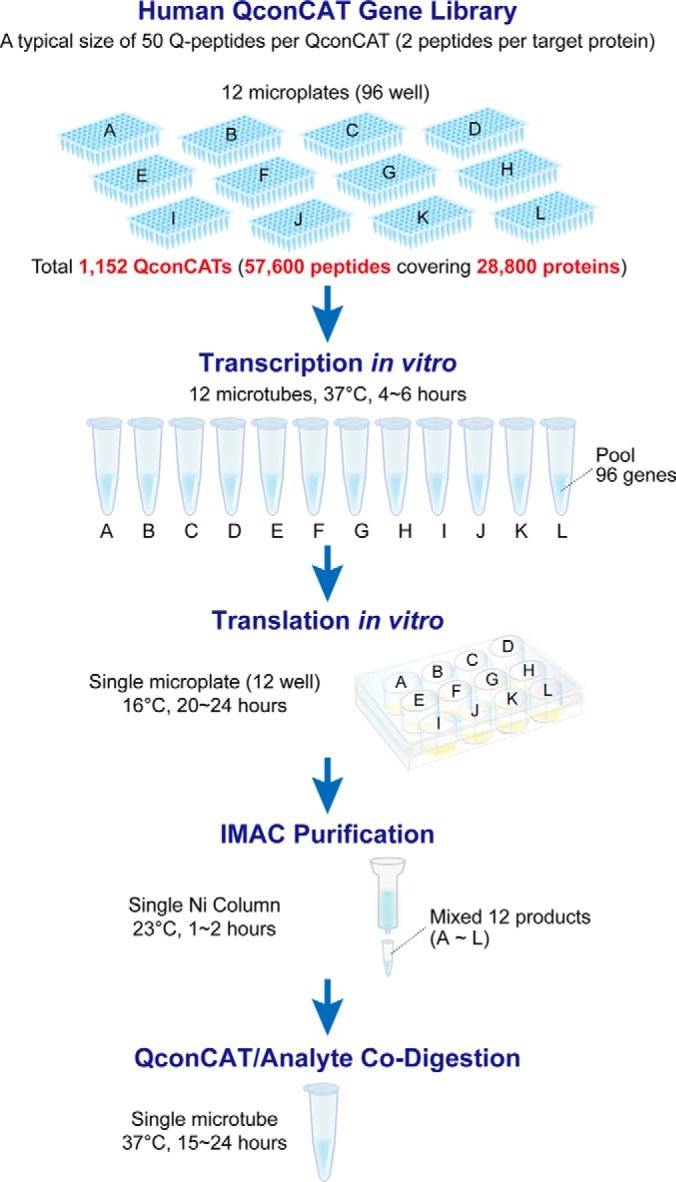
**A strategy for a human MEERCAT project.** A library of ∼1100 QconCAT template DNAs could be assembled in just 12 96-well microplates. In principle, the DNA template in each plate could then be pooled (adjusting DNA to alter the relative abundance of each QconCAT if required), transcribed and translated in a single 12-well microplate. One the QconCATs have been designed and synthesized, the entire set of QconCATs, independently tagged for quantification, could be prepared in a few days. At a typical QconCAT size of 50 peptides, this would yield a resource of over 57,000 quantified peptides, covering nearly 30,000 proteins; more than adequate to cover the entire human proteome.

How would a MEERCAT library be established for quantification of, for example, the human proteome? Setting a target of over 25,000 proteins/variants, it is perfectly feasible to contemplate the design of a 12 by 96 panel of QconCATs (Fig. 7). These would be available as plasmids that are readily propagated and distributed to any interested laboratory. Template DNA could then be amplified from the plasmid to drive transcription, or transcription could be driven directly using the plasmid as a template. Most usefully, the delivery of the plasmids could be driven by experimental design, such that individual laboratories would be able to request a coexpression reaction mixture to create an entire set of Q-peptides for their system. In total this panel, comprising 1152 plasmids, would encode ∼56,000 peptides, and thus, at a ratio of two peptides per target protein, would permit the absolute quantification of the entire human proteome. Even in the absence of robotic support, the synthesis of the entire panel of peptides could be complete within a matter of days, a phenomenal gain over the traditional route of construction of QconCATs or any other type of standard.

Although construction of this resource would be technically straightforward, a significant issue is that of peptide selection. In the quantification of *S. cerevisiae* proteins, we adopted two peptides per protein as a compromise between cost and analytical rigour. This allowed us to address the frequency with which two peptides disagreed—for those proteins for which we obtained two quantification values from two sibling peptides, the median log_2_ ratio between the two peptides was 0.54 and 70% of all sibling pairs yielded quantification values that were within a 2-fold difference (13). Selection of suitable Q-peptides can be directed by some obvious filters like those used previously (30). Ease of excision of each Q-peptide from the standard or the natural protein is a key factor that switches quantification from being stoichiometrically driven to being kinetically driven. Even if the Q-peptide and natural cognate are retained within the same primary sequence context (15), some sequences would be slow to digest, and these should be avoided. In addition, peptides containing amino acids prone to ectopic modification, such as methionine residues, should be excluded, as the signal splitting between the oxidized and nonoxidized variants of the peptides not only reduces sensitivity but is also unpredictable. Lastly, peptides should be selected based on their chromatographic behavior (narrow elution profile, retained by the stationary phases that are used routinely) and mass spectrometric behavior (clean and intense precursor ions, generation of consistent and high intensity fragment ions). It is intuitively obvious that peptides harboring known post-translational modifications should be avoided, as these can compromise quantification. It may also be possible to capitalize on the wealth of peptide data that is present in ProteomeExchange (31), GPM (32), PASSEL with Peptide Atlas (33) and SRMAtlas (34). The last, for example, contains over 158,000 SRM transitions that would inform QconCAT design. Similarly, the Proteome Tools project has already synthesized over one third of a million tryptic peptides, with a target of one million (35). In both instances, the peptides are proteotypic (unique to specific proteins) and further work would be required to discover which of these are quantotypic. Lastly, it may be increasingly possible to use such resources to develop predictive algorithms that assess optimal candidate peptides for quantification. However, it has rarely been formally established that such peptide data (observed or predicted) refer to true quantotypic peptides that can be used as surrogates for quantification. This may require a higher level of refinement as the resource is developed.

A final decision that must precede a “human MEERCAT” project relates to the strategy for clustering target proteins within QconCATs. There are two very different strategies. The first is based on an abundance clustering, such that proteins targeted by a QconCAT have similar biological expression levels. This raises the possibility of quantification in a single LC-MS/MS run, as standards and analytes would yield similar signals. However, for the *S. cerevisiae* project, QconCATs were designed to map specific pathways, reasoning that this clustering would be of greater value to other investigators. In this instance, subgroups of QconCATs targeting any pathway or functional class could be clustered by abundances, but it is more likely that repeated runs would be needed to titrate each standard and analyte signal such that they were within two or three orders of magnitude of each other. This consideration is driven in part by the dynamic range performance of the mass spectrometer being used, but also by the intensity of the “light” signal derived from the “heavy” QconCAT; as described above, synthesis *in vitro* allows better labeling than expression *in vivo*.

Using a model such as we describe here (Fig. 7), the synthesis of 1152 (12 × 96) QconCAT plasmids could be readily achieved for around Σ300,000 (less than $400,000), using current commercial rates for DNA synthesis. This is less than the cost of a single, high grade, LC-MS/MS system suitable for peptide quantification. Further, we could anticipate additional and substantial economies of scale for a complete human (or other) MEERCAT project. These plasmids are essentially an infinite resource, readily shared and propagated, either selectively or as an entire library, an advantage over synthetic peptide libraries (35). Each recipient laboratory would then be responsible for transcription and translation reactions, an approach that can also offer flexibility in terms of scale (few groups would require milligram quantities of each QconCAT) and labeling strategy. The WGCFS system is commercially available, and can be readily deployed by any reasonably well-equipped laboratory. The removal of any requirement for bacterial growth and the speed with which the WGCFS system can be completed also introduces technical and speed advantages.

As a final consideration, we advocate the WGCFS system as a system that is perfectly suited to exploratory studies using “designer proteins” for proteomics research. The ease of delivery of adequate quantities of labeled protein allows for cycles of exploration and refinement. Other concatamers have been developed for standardization of chromatography elution (36) and as hybrid standards for quantification of proteins by mass spectrometry and quantitative Western blotting (37). As more complex designs are conceived, it is increasingly attractive to be able to express such proteins free from issues of cellular toxicity/failure to express and proteolytic degradation.

## DATA AVAILABILITY

The QconCAT SRM files are deposited on PanoramaWeb (panoramaweb.org/labkey/meercat.url) and the DDA data files have been deposited to the ProteomeXchange Consortium via the PRIDE partner repository with the data set identifier PXD007949 and 10.6019/PXD007949.

## Supplementary Material

Supplemental Data

## Footnotes

^1^ The abbreviations used are:

WGCFS: wheat germ cell-free synthesis system
DDA: data directed acquisition
SRM: selected reaction monitoring
FP: fibrinopeptide
MS/MS: Tandem MS
LC-MS: liquid chromatography-mass spectrometry.
