# Supplementary material for: MEERCAT: Multiplexed Efficient Cell Free Expression of Recombinant QconCATs For Large Scale Absolute Proteome Quantification
Source: Mol Cell Proteomics. 2017 Oct 20;16(12):2169–83. doi: 10.1074/mcp.RA117.000284 (PMC5724179; doi:10.1074/mcp.RA117.000284)
Supplement: Supplemental Data [file supp_16_12_2169__index.html]

MEERCAT: MULTIPLEXED EFFICIENT CELL FREE EXPRESSION OF RECOMBINANT QconCATS FOR LARGE SCALE ABSOLUTE PROTEOME QUANTIFICATION — Multiplexed cell-free QconCAT biosynthesis — MEERCAT: Multiplexed Efficient Cell Free Expression of Recombinant QconCATs For Large Scale Absolute Proteome Quantification — Supplemental Data 

# MEERCAT: Multiplexed Efficient Cell Free Expression of Recombinant QconCATs For Large Scale Absolute Proteome Quantification

## Supplemental Data

- ORIGINAL Supplementary information - ORIGINAL Supplementary files referred to in main text.
- Supplementary Figures and Tables - REVISED Supplementary Figures and Tables
- fasta protein sequences - QconCat and level 2 Qconcat sequences used in this study
