# Supplementary material for: MEERCAT: Multiplexed Efficient Cell Free Expression of Recombinant QconCATs For Large Scale Absolute Proteome Quantification
Source: Mol Cell Proteomics. 2017 Oct 20;16(12):2169–83. doi: 10.1074/mcp.RA117.000284 (PMC5724179; doi:10.1074/mcp.RA117.000284)
Supplement: Supplemental Data [file supp_RA117.000284_4908_1_supp_13280_5x5v58.pdf]

Supplementary Figures for

MEERCAT: MULTIPLEXED EFFICIENT CELL FREE EXPRESSION OF RECOMBINANT QconCATS

FOR LARGE SCALE ABSOLUTE QUANTIFICATION OF PROTEOMES

Nobuaki Takemori, Ayako Takemori, Yuki Tanaka, Yaeta Endo, Jane L Hurst, Guadalupe Gómez-Baena, Victoria M Harman and Robert J Beynon

---

Supplementary Figure 1 | Detailed workflow for cell-free synthesis of QconCATs in vitro

Detailed workflow for the transcription and translation reactions for QconCAT synthesis. The final stages are for small, medium and large scale synthesis reactions

Transcription *in vitro*

1. Prepare a master mix for transcription *in vitro*

Add the following components to each 1.5 mL microtube

Component	Small-scale rxn	Middle-scale rxn	Large-scale rxn
Water, nuclease-free	4.44 µL	22.2 µL	111 µL
5 x Transcription Buffer [1]	2.5 µL	12.5 µL	62.5 µL
25 mM NTP Mix [1]	1.25 µL	6.25 µL	31.25 µL
80 U/µL RNase inhibitor [1]	0.16 µL	0.8 µL	4 µL
80 U/µL SP6 RNA Polymerase [1]	0.16 µL	0.8 µL	4 µL

[1] Reagents included in WEPRO8240H Expression kit (CellFree Sciences, Matsuyama, Japan).

2. Add template DNA

Add the template DNA solution to each tube

	PCR generated template (0.25 µg/µL)			Plasmid DNA (1 µg/µL)		
Component	Small rxn	Middle rxn	Large rxn	Small rxn	Middle rxn	Large rxn
Template DNA	4 µL	20 µL	100 µL	1 µL	5 µL	25 µL
Water, nuclease-free				3 µL	15 µL	75 µL

3. Incubate reactions (for 4-6 hours at 37°C)

Translation *in vitro*

1. Prepare translation mixture

Add the following components to each 1.5 mL microtube

Component	Small rxn	Middle rxn	Large rxn
mRNA	12.5 µL	62.5 µL	312.5 µL
Water, nuclease-free	4.07 µL	20.35 µL	101.75 µL
4x SUB-AMIX® [1, 2]	6.88 µL	34.4 µL	172 µL
Creatin Kinase (20 µg/µL) [1]	0.05 µL	0.25 µL	1.25 µL
Wheat germ extract (WEPRO®8240H) [1]	12.5 µL	62.5 µL	312.5 µL
400 mM $^{13}C_6/^{15}N_4$ Arg	2 µL	10 µL	50 µL
400 mM $^{13}C_6/^{15}N_2$ Lys	2 µL	10 µL	50 µL

[1] Reagents included in WEPRO8240H Expression kit (CellFree Sciences, Matsuyama, Japan).
[2] The mixture of heavy labeled 2 amino acids ($^{13}C_6$,$^{15}N_2$-Lys; $^{13}C_6$,$^{15}N_4$-Arg) and light labeled 18 amino acids.

2. Prepare 1xSUB-AMIX®

Component	Small rxn	Middle rxn	Large rxn
1x SUB-AMIX® (2.5 mM each of 20 AAs)	200 µL	1000 µL	5000 µL

3. Bi-layer reaction set-up

Make bi-layer with the translation mix in the lower layer and
1 x SUB-AMIX in the upper layer as illustrated below.

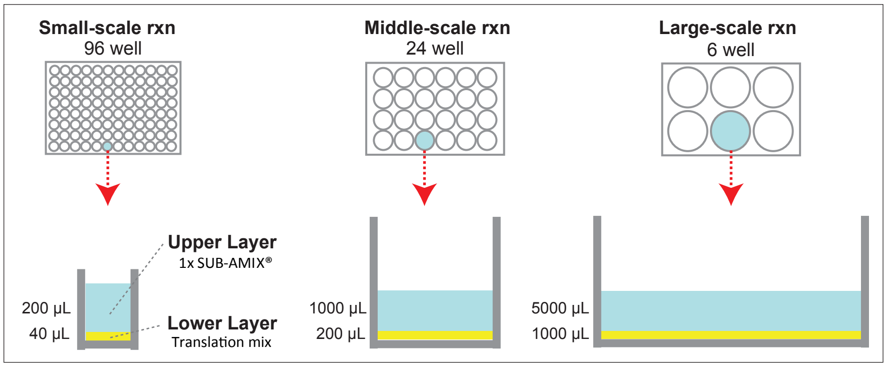

4. Incubate reactions (for 20-24 hours at 16°C)

Supplementary Figure 2 | Coverage maps for QconCATs expressed by cell-free synthesis

For a series of 12 QconCATs, 11 of which were not expressable in E coli, we completed expression in the cell-free system, and performed DDA on the purified protein. For each protein, peptides that were observed in the LC-MS/MS analysis are highlighted in coloured boxed, scaled green/yellow/red to reflect the relative peptide score. Missing peptides are highlighted with single lines. In all QconCATs, the N-terminal Glu-fib standard peptide at the N-terminus $[M+2H]^{2+}$ of 785.83 and the C-terminal hexahistidine tag $[M+2H]^{2+}$ 705.33 were detected, confirming the intactness of each QconCAT.

Protease: Trypsin [R-X, K-X, not R-P, K-P]

Substrate:CC001[571 amino acids, approx 62.3kDa]

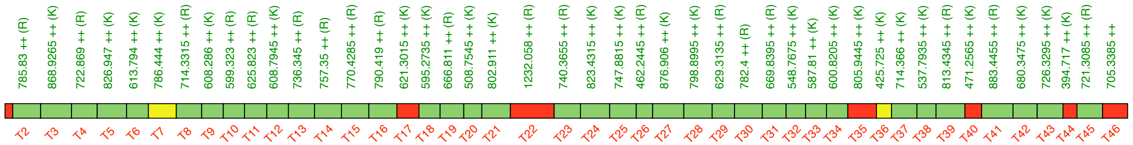

Substrate:CC024[688 amino acids, approx 75.8kDa]

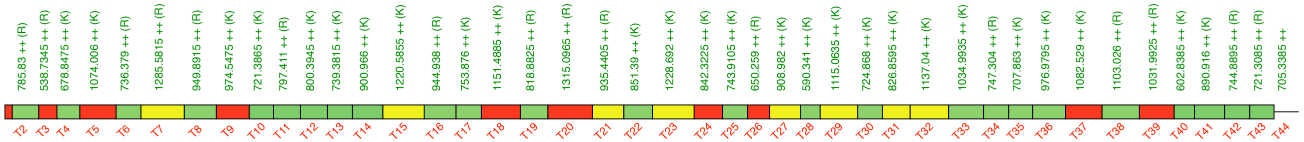

Substrate:CC039[724 amino acids, approx 79.6kDa]

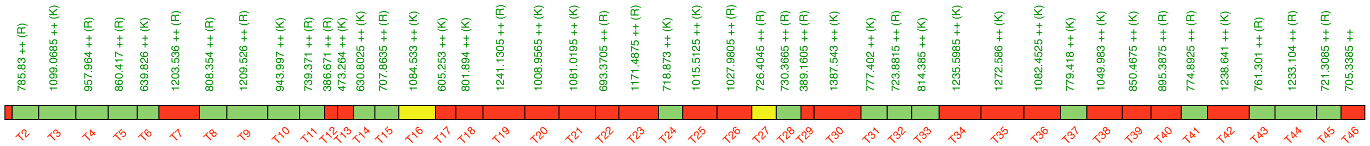

Substrate:CC046[765 amino acids, approx 82.4kDa]

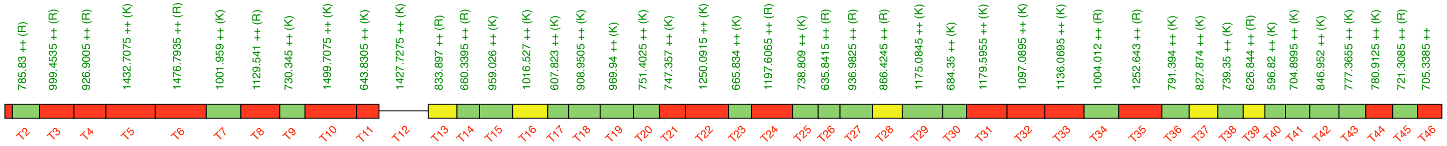

Substrate:CC057[642 amino acids, approx 70.9kDa]

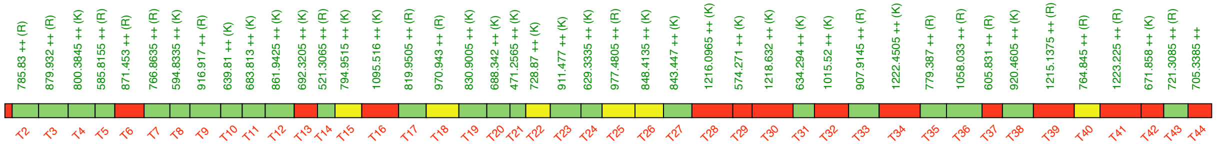

Substrate:CC062[752 amino acids, approx 83.8kDa]

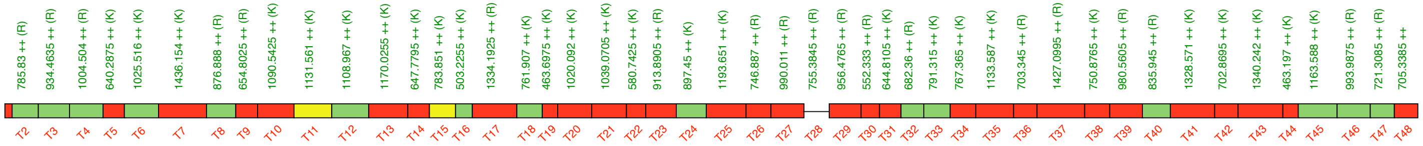

Substrate:CC064[690 amino acids, approx 77kDa]

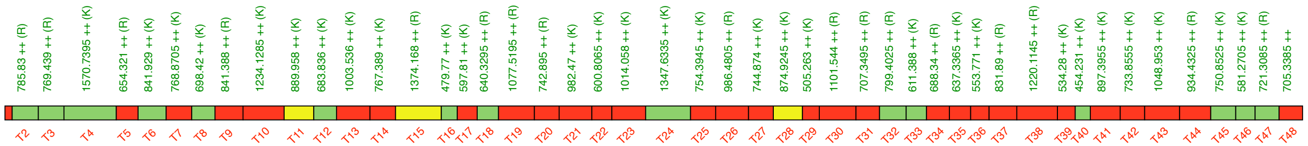

Substrate:CC066[708 amino acids, approx 78.7kDa]

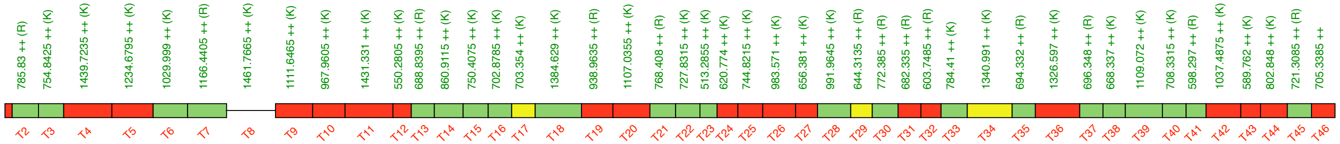

Substrate:CC069[689 amino acids, approx 75.9kDa]

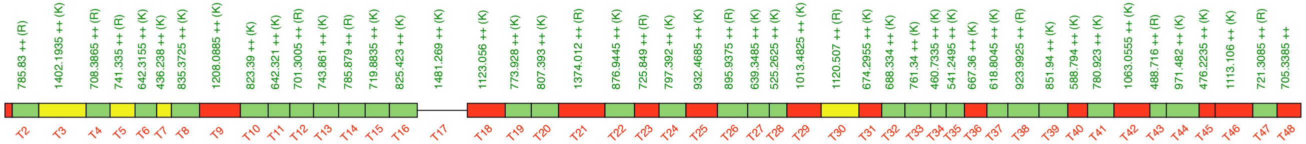

Substrate:CC069[700 amino acids, approx 76.8kDa]

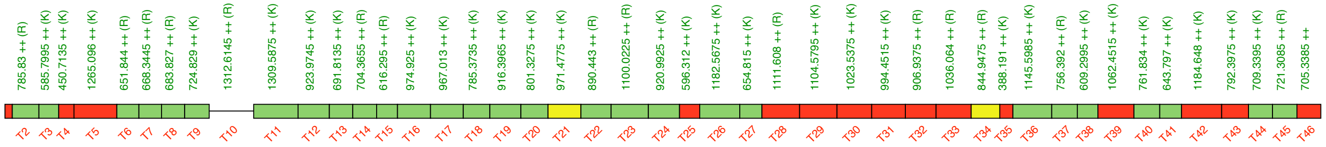

Substrate:CC099[581 amino acids, approx 63.5kDa]

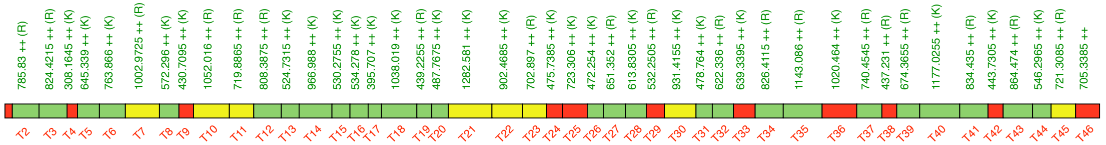

Substrate:CC103[552 amino acids, approx 60.7kDa]

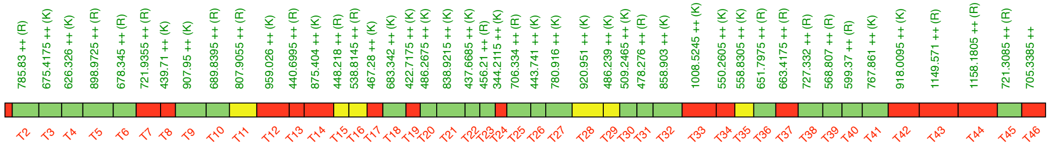

Supplementary Figure 3 | Absolute quantification of wheat germ cell-free synthesized QconCATs

Absolute quantification of synthesized QconCATs (those that had previously failed in *E. coli*, expect for a positive control, #1) in WGCFS was performed using stable isotope dilution mass spectrometry (panel A). A mixture of tryptic digests of $^{13}C/^{15}N$-labeled QconCAT (Heavy) was mixed with GluFib peptide (Light, 10 pmol) and subjected to LC-SRM analysis (panel B). Quantification of synthesized QconCAT abundance is based on the peak area ratios of the light (internal standard) and the heavy (tryptic peptide derived from synthesized QconCAT) form of GluFib peptide (panels C and D).

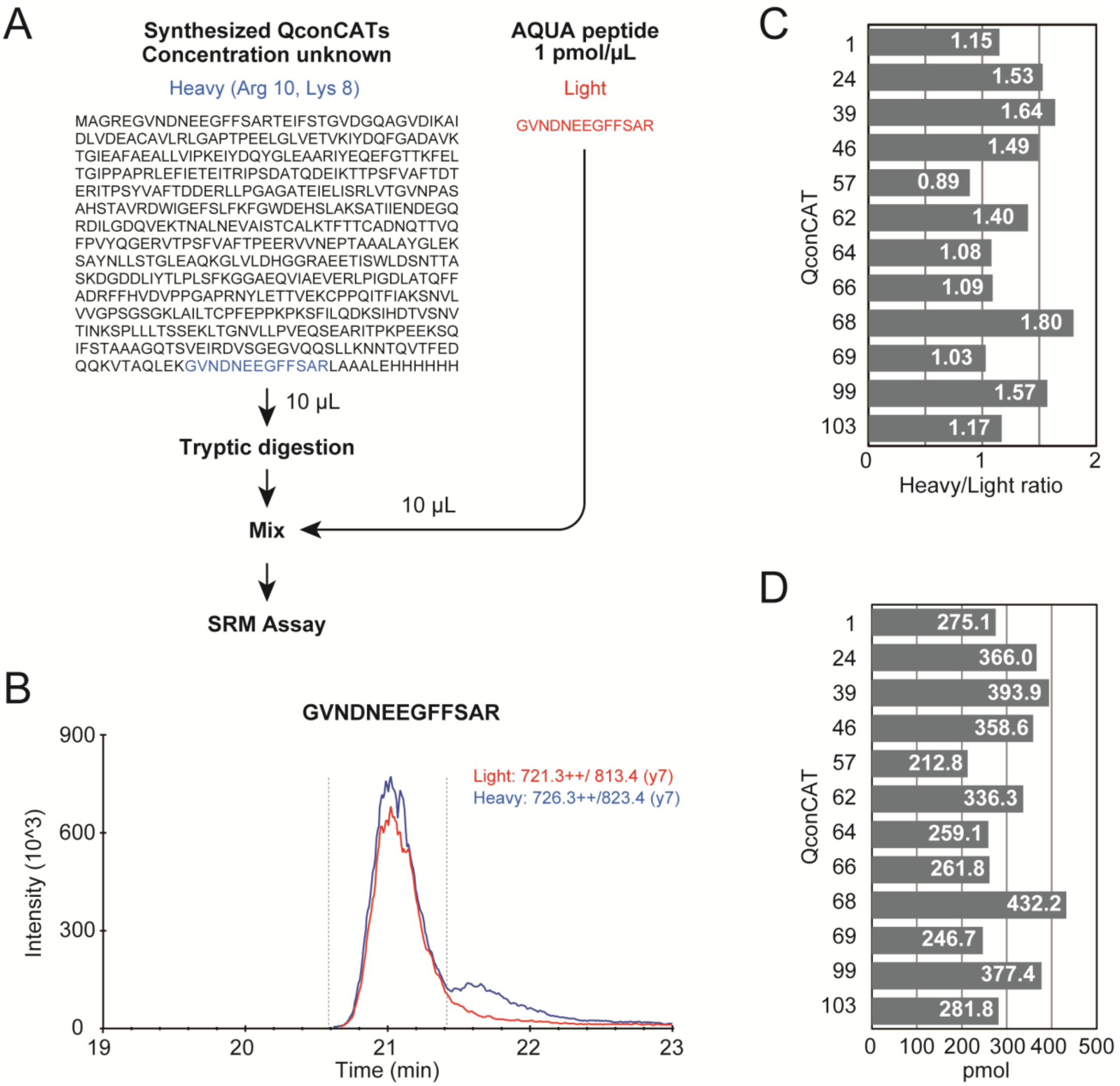

Supplementary Figure 4 | Estimated amount of 12 QconCAT components co-synthesized in WGCFS

The estimation of the amount of each QConCAT component obtained by simultaneous synthesis of multiple QconCAT (Figure 2) was performed using SRM assay. Two different target peptides (panels A and B) were used for each QconCAT in the SRM assay.

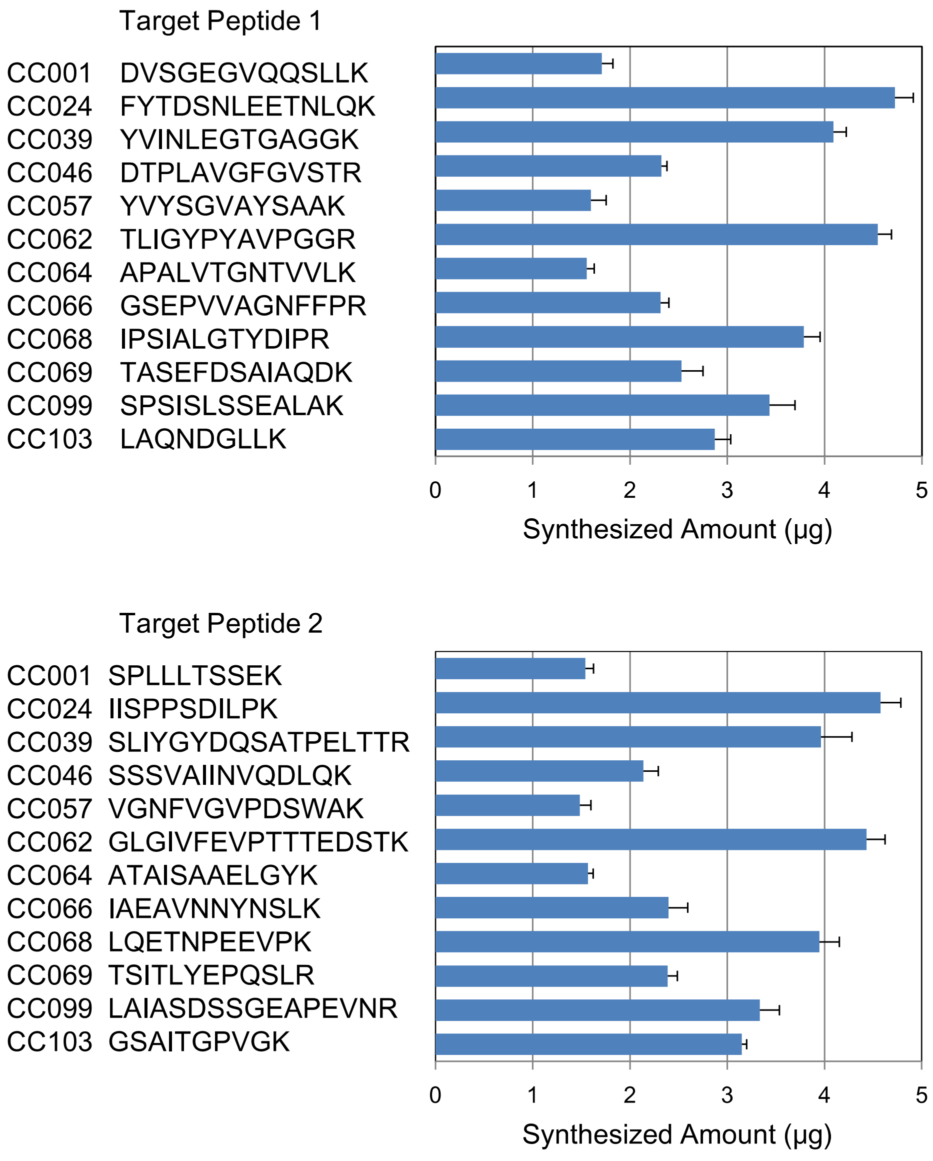

Supplementary Figure 5 | Cell-free synthesis of QconCAT series developed for quantification of yeast proteome

Each QconCAT was independently synthesized on a small scale (240 µL). Unpurified samples were separated using a 4-12% NuPAGE gel. Representative gel separation images visualized by CBB staining are shown. Asterisk: QconCAT band identified by mass spectrometry. For two QconCATs (32, 12v2) there were problems of template PCR product generation and synthesis could not be assessed,

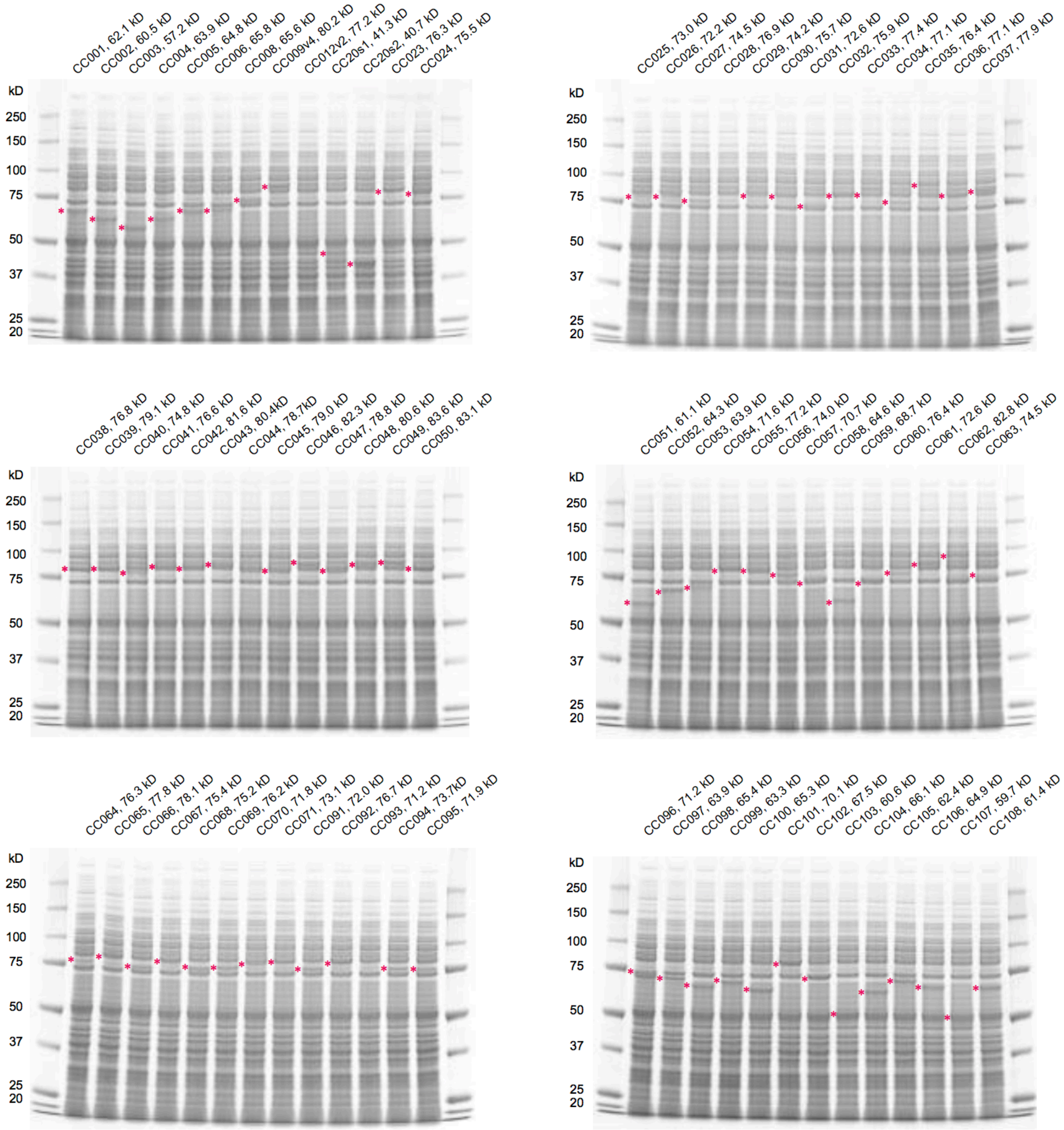

Supplementary Figure 6 | Large scale synthesis of QconCAT by MEERCAT

Using the MEERCAT approach (6 mL scale) shown in Supplementary Fig. 1, 150 types of small QconCATs were synthesized simultaneously. The synthesized QconCATs were verified by gel electrophoresis and mass spectrometry. Panel A shows the SDS-PAGE image (CBB staining) of the sample after His tag purification. The purified sample was also subjected to DDA analysis by LC-MS/MS after trypsin digestion. Panel B shows the sequence coverage of the identified 149 QconCATs.

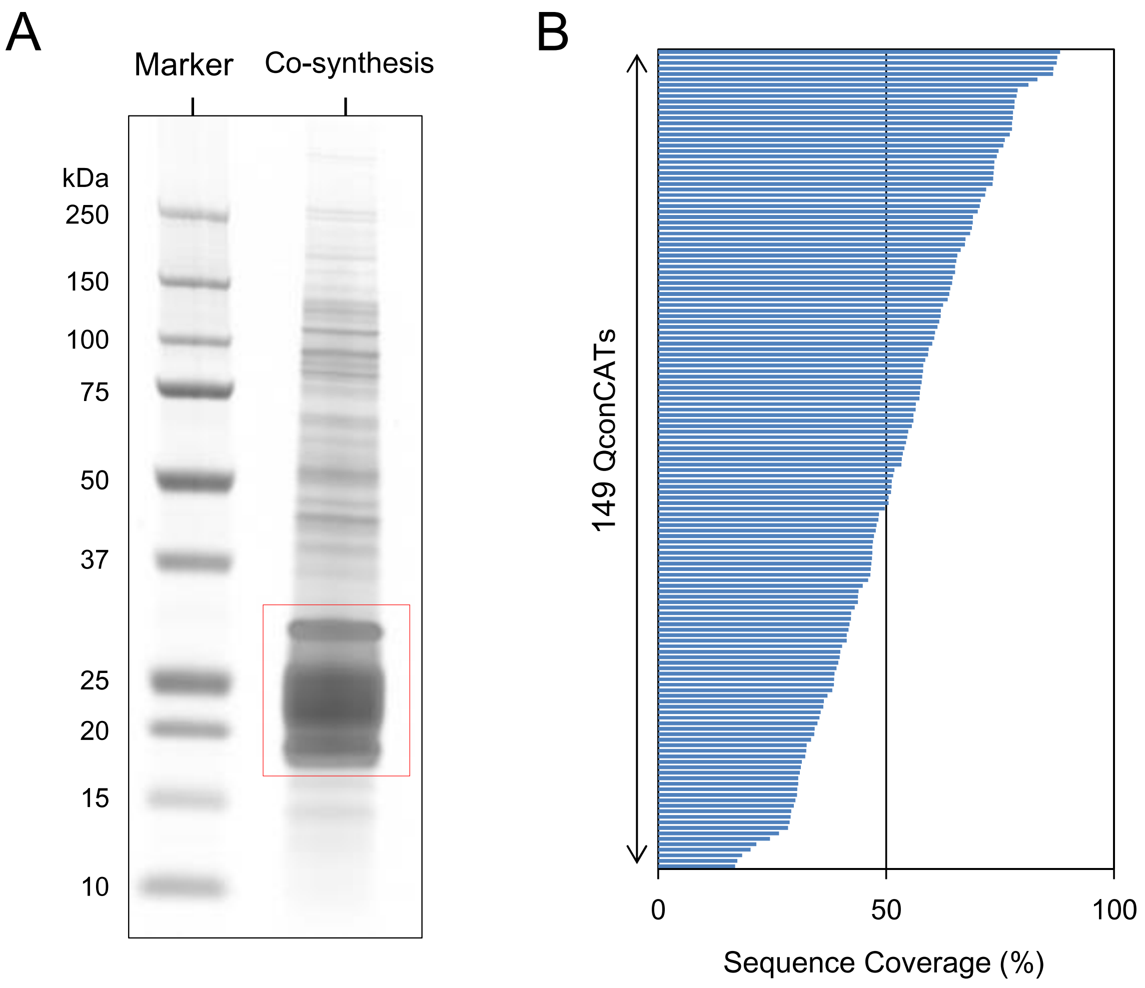

Supplementary Figure 7 | Quantitative strategy of absolute quantities of each QconCAT component in co-synthesis reaction
Introducing of re-useable tag sequences (panel A) allows encoding of 100 QconCATs for quantification. For absolute quantification of each QconCAT, accurately quantified 'second order' QconCATs, which are optimized for rapid and effective proteolysis and including all 100 Tags, are used as internal standards for MS quantification. Panel B shows representative second order QconCAT sequence encoding Tag1A to 1J.

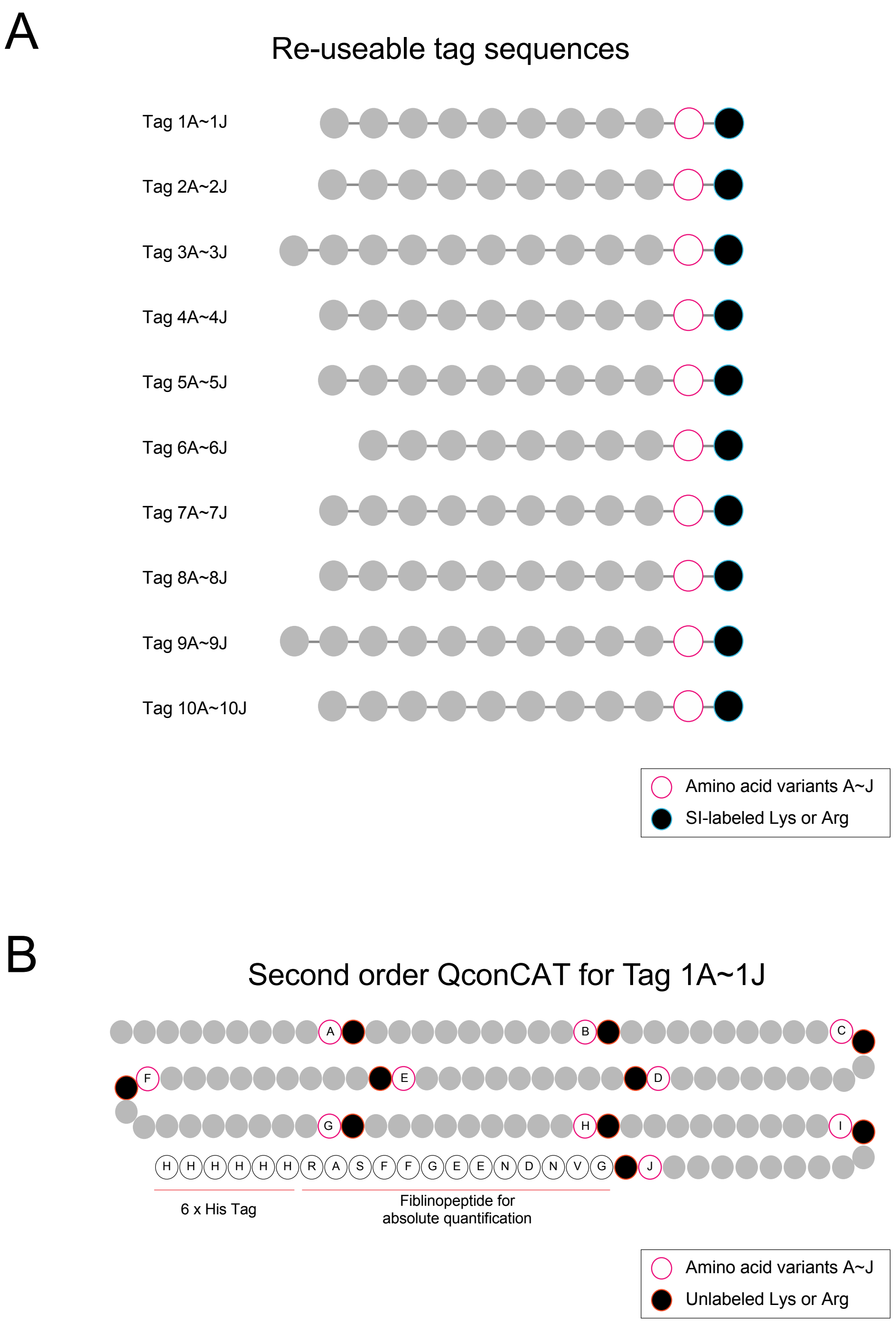

Supplementary Figure 8 | Workflow comparison of bacterially expressed QconCATs and cell-free synthesized QconCATs

The workflows are expressed in terms of the time required for each step, using a standard workflow for QconCATs used in the yeast COPY project and the cell free synthesis protocol used in this paper.

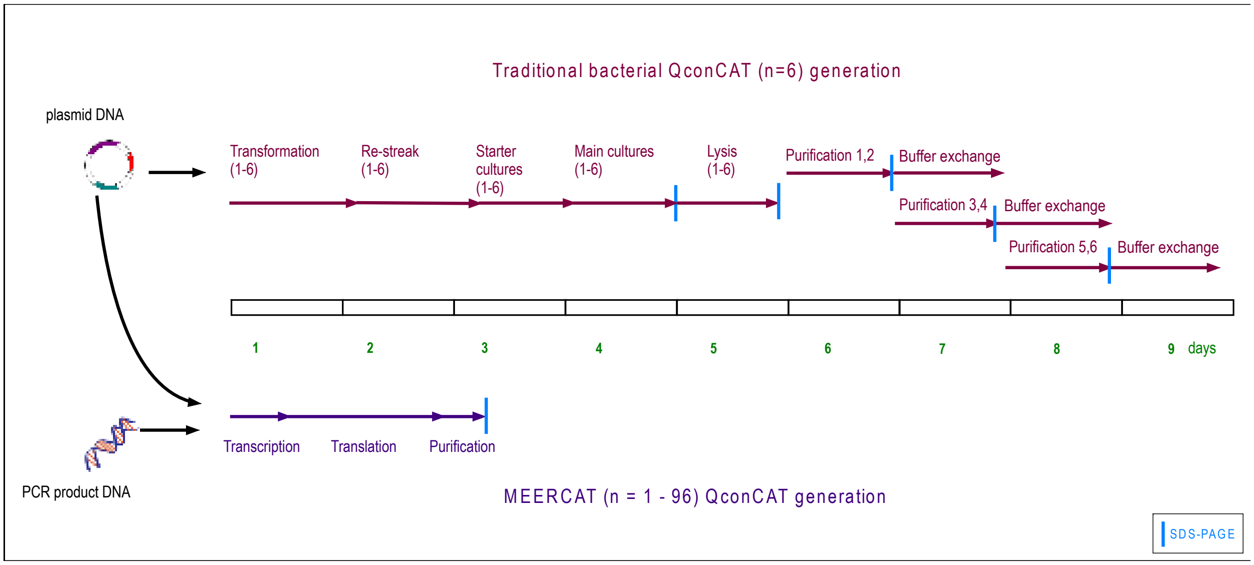

Supplementary Table 1 | Estimation of labelling efficiency for QconCATs synthesized *in vitro*

For one QconCAT, the labelling efficiency for assessed across multiple peptides by calculation of the peak area of the labelled and unlabeled forms, either for lysine labelling (top) or arginine labelling (bottom).

Protein	Peptide Sequence (QconCAT 001)	Peak Area (Light)	Peak Area (Heavy)	$^{13}C_6/^{15}N_2$-Lys Incorporation Efficiency (%)
QconCAT 001	TEIFSTGVDGQAGVDIK	5.3E+04	8.5E+06	99.38
	LGAPTPEELGLVETVK	4.5E+04	5.1E+06	99.11
	IYDQFGADAVK	9.5E+04	2.4E+07	99.61
	IYEQEFGTTK	6.6E+04	2.0E+07	99.67
	IPSDATQDEIK	6.6E+03	2.1E+06	99.69
	VVNEPTAAALAYGLEK	1.4E+04	5.4E+06	99.74
	SAYNLLSTGLEAQK	6.4E+04	2.7E+07	99.76
	NYLETTVEK	3.2E+04	1.6E+07	99.80
	SNVLVVGPSGSGK	5.2E+04	1.7E+07	99.69
	SIHDTVSNVTINK	2.8E+03	1.3E+06	99.78
	SPLLLTSSEK	7.3E+04	3.6E+07	99.79
			Average	99.6±0.2

Protein	Peptide Sequence (QconCAT 001)	Peak Area (Light)	Peak Area (Heavy)	$^{13}C_6/^{15}N_4$-Arg Incorporation Efficiency (%)
QconCAT 001	EIYDQYGLEAAR	7.2E+04	1.5E+07	99.52
	FELTGIPPAPR	3.8E+04	4.4E+07	99.91
	LEFIETEITR	6.6E+04	1.3E+07	99.49
	TTPSFVAFTDTER	8.5E+04	1.4E+07	99.41
	ITPSYVAFTDDER	5.6E+04	1.1E+07	99.50
	LLPGAGATEIELISR	2.7E+04	4.0E+06	99.31
	GLVLDHGGR	6.6E+03	3.1E+06	99.79
	GGAEQVIAEVER	6.9E+04	2.8E+07	99.75
	LPIGDLATQFFADR	5.0E+03	1.4E+06	99.64
			Average	99.6±0.2
